# Influence of graft composition in patients with hematological malignancies undergoing ATG-based haploidentical stem cell transplantation

**DOI:** 10.3389/fimmu.2022.993419

**Published:** 2022-09-15

**Authors:** Ran Zhang, Xuan Lu, Liang V. Tang, Huafang Wang, Han Yan, Yong You, Zhaodong Zhong, Wei Shi, Linghui Xia

**Affiliations:** ^1^ Department of Hematology, The First Affiliated Hospital of Zhengzhou University, Zhengzhou, China; ^2^ Institute of Hematology, Union Hospital, Tongji Medical College, Huazhong University of Science and Technology, Wuhan, China

**Keywords:** haploidentical hematopoietic stem cell transplantation, graft composition, G-CSF-mobilized BM cells, G-CSF-mobilized PBSCs, total nucleated cells

## Abstract

To determine the influence of graft composition in haplo-HSCT, we summarized the long-term consequences of 251 consecutive transplantations from haploidentical donors. For donor-recipient HLA3/6-matched setting, 125 cases used G-CSF-mobilized BM and PBSCs mixtures, while 126 cases only used G-CSF-mobilized PBSCs in HLA4/6-matched transplantation. On the one hand, we wanted to explore the effect of harvests (CD34+ cells and TNCs dosages) on transplantation outcome in the context of haplo-HSCT no matter HLA4/6 or HLA3/6-matched setting. On the other hand, for patients using G-CSF-mobilized BM and PBSCs combination in HLA3/6-matched setting, we attempted to analyze whether TNCs or CD34+ cells from G-CSF-mobilized BM or G-CSF-mobilized PBSCs play the most paramount role on transplantation prognosis. Collectively, patients with hematologic malignancies receiving G-CSF-primed BM and PBSCs harvests had comparable consequences with patients only receiving G-CSF-mobilized PBSCs. Moreover, when divided all patients averagely according to the total amount of transfused nucleated cells, 3-year TRM of the intermediate group (13.06-18.05×10^8^/kg) was only 4.9%, which was remarkably reduced when compared to lower and higher groups with corresponding values 18.3%, 19.6% (*P*=0.026). The 3-year probabilities of OS and DFS of this intermediate group were 72.6% and 66.5%, which were slightly improved than the lower and higher groups. Most importantly, these data suggest that the transfused nucleated cells from G-CSF-primed BM above than 5.20×10^8^/kg could achieve remarkably lower TRM in haplo-HSCT receiving G-CSF-mobilized BM and PBSCs harvests. These encouraging results suggested that we could improve the efficacy of haplo-HSCT by adjusting the component and relative ratio of transfused graft cells. Nevertheless, the above findings should be confirmed in a randomized prospective comparative research with adequate follow-up.

## Introduction

Nowadays, allogeneic hematopoietic stem cell transplantation (allo-HSCT) is the exclusively potential curative treatment for patients with hematological malignancies. In China, haploidentical HSCT (haplo-HSCT) has been widely accepted as an alternative approach for treating patients with high-risk hematological diseases who do not own a human leukocyte antigen (HLA) identical sibling donor and urgently need transplantation (1–3).

More recently, we have successfully established an original haplo-HSCT protocol using idarubicin (IDA) intensified conditioning regimen and incorporation of antithymocyte globulin (ATG) and basiliximab as graft-versus-host disease (GVHD) prophylaxis. In our system, we adopted the mixture of granulocyte colony-stimulating factor (G-CSF)-mobilized bone marrow (BM) and peripheral blood stem cells (PBSCs) and an overall dose of 9mg/kg ATG in HLA3/6-matched setting. While in HLA4/6-matched transplantation, we only used G-CSF-primed PBSCs and a total dosage of 6mg/kg ATG. Remarkably, our encouraging haplo-HSCT protocol could remarkably enhance the survival in high-risk acute leukemia patients with considerably lower relapse incidence and acceptable transplantation-related mortality (TRM), and could be considered as a preferable therapeutic option for high-risk population (4). Surprisingly, there was no significant discrepancy between HLA3/6-matched and HLA4/6-matched setting regardless of primary recurrence, GVHD and survival. Therefore, we attempted to broaden the utilization of G-CSF-mobilized PBSCs to HLA3/6-matched haplo-HSCT. Subsequently, this specific background raises the question of whether the supplement of G-CSF-mobilized BM cells to G-CSF-mobilized PBSCs could bring favorable therapeutic efficacy in the setting of haplo-HSCT.

Each graft source has its distinctive constitutions, such as the amount of CD34+ cells, diverse T cell and natural killer cell subpopulations and, probably, other cytokine as well as chemokine components, which might influence the time frame and kinetics of hematopoietic reconstitution, occurrence of acute and chronic GVHD and relapse rate, and thus may indicate the choice of grafts under difference circumstances (5, 6). Substantial controversy still exists respecting to the best graft source and the effect of graft composition on the occurrence of GVHD, disease control, prognosis and other crucial clinical outcomes (7–9). Over the past decade, PBSCs mobilized by G-CSF have contributed to the worldwide adoption of HSCT and replaced BM as the prominent stem cell source in allo-HSCT due to rapid hematopoietic engraftment kinetics, a reduced risk of graft failure, possibly a lower incidence of recurrence, easier graft procurement, and lower risk of donor complications (10). Nevertheless, recent researches indicated that G-CSF-mobilized BM had similar engraftment but obviously lower morbidity and mortality of GVHD in comparison to G-CSF-primed PBSCs (11).

Therefore, in the current research, we devoted to testifying the outcome using G-CSF-primed PBSCs and BM combination grafts in hematological malignancies patients undergoing haplo-HSCT, and compared the results with a contemporary cohort of cases only receiving G-CSF-mobilized PBSCs. In addition, until now, it is still controversial about the effect of harvests (CD34+ cells and total nucleated cells (TNCs) dosages) and the optimal thresholds of the transplanted harvests in haplo-HSCT. Thus, identification of the most appropriate threshold of the transplanted harvests is warranted as a potential strategy to improve prognosis in haplo-HSCT platform.

## Patients and methods

### Patients

A total of two hundreds and fifty-one consecutive patients who were diagnosed with hematological malignancies and underwent the first allo-HSCT from an HLA-haploidentical donor between August 2014 and April 2021 were enrolled in the present study. This time period was determined to allow a minimum follow-up of 12 months after allo-HSCT for surviving patients. All patients and donors provided written informed consent, in accordance with the Declaration of Helsinki. The studies involving human participants were reviewed and approved by the Institutional Review Board on Medical Ethics at Tongji Medical College of Huazhong University of Science and Technology. The characteristics and details of patients and transplantations were delineated as in **Table 1**. The closeout date for analyses was April 30, 2022.

**Table 1:** Patient and transplant characteristics.

Characteristics	G-CSF-primed BM and PBSCs	G-CSF-primed PBSCs	*P*-value
No. of patients	125	126	
Age, median (range), year	29 (8-56)	28 (6-59)	0.657
Gender			0.662
Male	72 (57.6%)	76 (60.3%)	
Female	53 (42.4%)	50 (39.7%)	
Underlying disease, n (%)			0.600
AML	61 (48.8%)	64 (50.8%)	
ALL	47 (37.6%)	50 (39.7%)	
MDS	17 (13.6%)	12 (9.5%)	
Risk classification, n (%)			0.320
Standard risk	27 (21.6%)	34 (27.0%)	
High risk	98 (78.4%)	92 (73.0%)	
HCT-CI, n (%)			0.460
0	50 (40.0%)	43 (34.1%)	
1-2	48 (38.4%)	48 (38.1%)	
>2	27 (21.6%)	35 (27.8%)	
HLA typing			<0.001
HLA4/6-matched	18 (14.4%)	97 (77.0%)	
HLA3/6-matched	107 (85.6%)	29 (23.0%)	
Donor/recipient CMV status			0.749
Neg/neg	58 (46.4%)	50 (39.7%)	
Neg/pos	26 (20.8%)	31 (24.6%)	
Pos/neg	23 (18.4%)	25 (19.8%)	
Pos/pos	18 (14.4%)	20 (15.9%)	
Donor-recipient relationship			0.360
Parent donor	50 (40.0%)	43 (34.1%)	
Child donor	26 (20.8%)	33 (26.2%)	
Sibling donor	47 (37.6%)	50 (39.7%)	
Lateral relative donor	2 (1.6%)	0 (0)	
Conditioning intensity			0.347
Intensified regimen	93 (74.4%)	87 (69%)	
MAC	32 (25.6%)	39 (31%)	
Median nucleated cells, ×10^8^/kg (range)	16.08 (2.14-48.35)	14.62 (3.83-41.03)	0.920
Median CD34+ cells, ×10^6^/kg (range)	6.05 (1.65-21.51)	6.29 (2.57-25.1)	0.567
Median follow-up for survivors (months, range)	39 (22-91)	41 (22-92)	0.127

AML, acute myeloid leukemia; ALL, acute lymphoblastic leukemia; MDS, myelodysplastic syndrome; HLA, human leukocyte antigen; MAC, myeloablative conditioning; G-CSF, granulocyte colony-stimulating factor; BM, bone marrow; PBSCs, peripheral blood stem cells; HCT-CI, hematopoietic stem cell transplantation-comorbidity index; CMV, cytomegalovirus.

### HLA matching and stem cell harvesting

HLA match was defined on the basis of 6/6 serological match at HLA-A, B and -DR loci. Among potential family members, HLA-A and HLA-B was typed by intermediate-resolution DNA techniques, whereas HLA-DRB1 was performed through high-resolution DNA techniques. The recipient of haplo-HSCT was transplanted from a family member who was matched at one HLA haplotype with the patient but differed to a variable extent among the HLA-A, B and DR antigens of the unshared haplotype. Donors were preferentially selected on the basis of HLA-matched loci, donor specific antibodies (DSA), younger age, male gender, and better performance conditions.

Recombinant human G-CSF (rhG-CSF, 8-10 μg/kg/d for 5 days) with subcutaneous administration was utilized to prime BM and/or peripheral blood stem cells. In the G-CSF-mobilized PBSCs alone cohort, G-CSF-mobilized PBSCs were infused into the recipient after collection without manipulation on the fourth and fifth day (on the sixth day if needed). Meanwhile, in the G-CSF-primed PBSCs and BM combination group, G-CSF-mobilized BM was harvested on fourth day, and G-CSF-primed PBSCs were collected on the fifth day (and on the sixth day if needed). The unmanipulated BM and PBSCs were transfused into the recipients after collection. For exploring the influence of graft contents on transplant outcomes in the whole population, the number of infused TNCs and CD34+ cells were stratified averagely into low, intermediate and high categories. Then, the G-CSF-mobilized BM and PBSCs cohort was classified into dichotomized variables according to cutoff levels of infused nucleated cells or CD34+ cells harvested from G-CSF-primed BM or G-CSF-mobilized PBSCs due to the limited numbers.

### Conditioning regimens

Generally, patients who were diagnosed with acute myeloid leukemia (AML) or myelodysplastic syndrome (MDS) used IDA-BUCY2 regimen or traditional BUCY2 regimen, while patients with acute lymphoblastic leukemia (ALL) received regimens of IDA-intensified total body irradiation (TBI)-CY or traditional TBI-CY, which were consistent with our previous reports (4, 12–14). ATG (Sanofi Aventis, Paris, France) was given to all patients as part of the conditioning regimen. Patients received at a total dosage of 6mg/kg (3 mg/kg per day, days -1 to 0) in donor-recipient HLA 4/6-matched haplo-HSCT, whereas in HLA 3/6-matched transplant, a total dosage of 9mg/kg ATG was used from day -4 to -2.

### GVHD prophylaxis

Prophylaxis against GVHD consisted of cyclosporine A (CsA), short-term methotrexate (MTX), mycophenolate mofetil and basiliximab. CsA at 2.5 mg/kg/day was initiated intravenously on day -1 with target levels of 150-250 ng/mL and switched to oral form when patients were able to tolerate. The administration of MTX was given to patients at 15 mg/m^2^ on day +1 and 10 mg/m^2^ on days +3, +6, and +11. Mycophenolate mofetil (7.5 mg/kg, twice daily) was administered orally from day -9, reduced to half-dose until day +30, finally discontinued on day +60. Basiliximab (20 mg by 40-minute i.v. infusion) was administered on day 0 and day +4. Decrement of immunosuppressants was based on the presence or absence of severe GVHD, infectious diseases, and relapse risks, starting at 2-4 months after allo-HSCT, aiming at cessation by approximately 6 months without manifestation of GVHD. aGVHD and cGVHD were diagnosed and graded according to the standard criteria (15, 16).

### Evaluation and monitoring of engraftment, MRD

Neutrophil recovery was defined as achieving an absolute neutrophil count higher than 0.5×10^9^/L for three consecutive days, and platelet recovery was defined as platelets higher than 20×10^9^/L without transfusions during the preceding seven days. Morphologic and cytogenetic analysis of BM using flow cytometry and qRT-PCR were scheduled for 1, 3, 6, 9, and 12 months and subsequently every 3 months during 12 to 24 months after allo-HSCT, as well as whenever clinically indicated. BM chimerism was evaluated at the same time through PCR amplification of short tandem repeats (STR).

### Supportive care

Supportive care after allo-HSCT, including the usage of empirical antibiotics, prophylaxis and therapy for cytomegalovirus (CMV), and hepatic veno-occlusive disease (HVOD) prevention were executed as our previous reports (4, 12–14).

### Statistics

Comparisons among different cohorts were analyzed by Chi-squared or Fisher’s exact tests for categorical variables and Mann-Whitney U test for continuous variables, respectively. The probabilities of hematopoietic reconstitution, acute and chronic GVHD, relapse and TRM were analyzed by the cumulative incidence estimator to accommodate competing risks. Incidences of time-dependent outcomes were calculated by the Kaplan-Meier method and compared by the log-rank test. For univariate analyses, continuous variables were categorized and the median used as a cutoff point. Cox proportional hazards model was applied to calculate the interaction between patient/graft characteristics and various transplant outcomes. Factors known to influence the outcome and factors associated with a *P-*value less than 0.10 with any endpoint in the univariate Cox analysis were eligible for inclusion in the multivariate Cox regression analysis. Statistical analyses were performed using SPSS 22.0 and GraphPad Prism 6. All statistical analysis was two-sided, and *P*-value less than 0.05 indicated statistical significance.

## Results

### Patients’ and transplant’s characteristics

A total of 251 patients were included in the present study, with 125 in G-CSF-primed BM and PBSCs group and 126 cases in G-CSF-mobilized PBSCs group, respectively. Characteristics of the patient and transplantation between two cohorts were summarized in **Table 1**. Except that higher proportion of donor-recipient HLA 3/6-matched in the G-CSF-mobilized BM and PBSCs group (85.6% *vs.* 23.0%, *P*<0.001), the two cohorts were well-balanced in terms of other parameters including patients’ age, gender, primary disease, risk classification, HCT-CI, donor-recipient relationship, donor-recipient CMV status, conditioning regimen, numbers of infused nucleated cells and CD34+ cells (all *P*>0.05). The median follow-up times for surviving cases were 39 months, 41 months, respectively (*P*=0.127).

### Engraftment and GVHD

Five patients in G-CSF-mobilized BM and PBSCs group and three in G-CSF-mobilized PBSCs group failed to acquire neutrophil reconstitution and subsequently died of lethal infection (4.0% *vs.* 2.4%, *P*=0.500), the others achieved hematopoietic reconstitution smoothly. Successful neutrophil recovery was achieved in G-CSF-mobilized BM and PBSCs cohort after a median of 11 days (range, 9 to 24), which was resemble to that in the G-CSF-mobilized PBSCs cohort with a median of 11 days (range, 8 to 20) (*P*=0.998). No remarkable difference was observed in the platelet engraftment between two cohorts (G-CSF-primed BM and PBSCs group, 13 days (range, 9 to 73); G-CSF-mobilized PBSCs group, 12 days (range, 10 to 60), *P*=0.504).

For the G-CSF-primed BM and PBSCs population, aGVHD occurred in 39 cases after a median period of 45 days post-transplantation. Among patients who developed aGVHD, 35 cases were I-II aGVHD, while four were III-IV aGVHD. It must be noteworthy that two I-II aGVHD and two III-IV aGVHD episodes occurred after utilizing donor lymphocyte infusion (DLI) because of primary recurrence. Thirty-one patients had only one organ involved (skin n=20, gut n=10, and liver n=1), whereas six patients had two organs involved and only two had three organs involved. While in G-CSF-mobilized PBSCs group, 32 patients developed aGVHD including 24 cases with I-II aGVHD and eight with III-IV aGVHD. And one I-II aGVHD as well as six cases with III-IV aGVHD occurred after the application of DLI. Twenty-three patients had one organ involved (skin n=13, gut n=7, and liver n=3), five cases had two organs involved and four had three organs involved. In patients receiving G-CSF-mobilized BM and PBSCs, I-IV aGVHD occurred at a median period of 43 days (range, 24-80) with the +100 days cumulative incidence of 30.1%, whereas in G-CSF-primed PBSCs group, the cumulative incidence at day +100 was 20.3% and the median interval was 46 days (range, 27-75) (hazard ratio (HR), 1.527; 95% confidence interval (CI): 0.915-2.547, *P*=0.105). 111 patients in G-CSF-primed BM and PBSCs cohort as well as 111 patients in G-CSF-mobilized PBSCs group were eligible to be evaluated for cGVHD. The cumulative incidence of total cGVHD at 2 years after allo-HSCT in G-CSF-mobilized BM and PBSCs cohort and G-CSF-primed PBSCs group were 30.3%, 26.5%, separately (HR, 1.244; 95% CI: 0.734-2.109, *P*=0.418). The cumulative incidence cGVHD at 3 years of two cohorts were 32.9%, 28.1%, respectively (HR, 1.263; 95% CI: 0.626-2.546, p=0.514).

### Relapse and TRM

**Figure 1:**
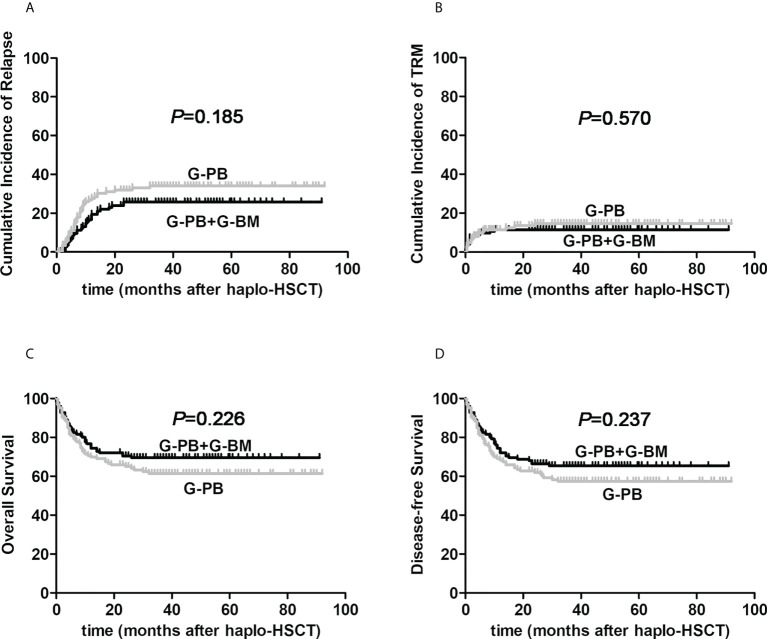
The influences of graft constituents on post-transplant outcomes of patients with hematological malignancies undergoing ATG-based haplo-HSCT. The black line represents patients the combination of G-CSF-primed BM and PBSCs (n=125), the gray line represents patients the combination of G-CSF-primed PBSCs (n=126). **(A)** relapse; **(B)** TRM; **(C)** Overall survival; **(D)** Disease-free survival.

As exhibited in the **Figure 1A**, the 3-year cumulative incidence of primary disease recurrence in G-CSF-mobilized BM and PBSCs cohort showed resemble result when compared with G-CSF-mobilized PBSCs group (26.5% *vs.* 34.7%, HR, 0.725; 95% CI: 0.451-1.166, *P*=0.185). In patients receiving G-CSF-primed BM and PBSCs grafts, 30 cases suffered relapse of primary disease at a median duration of 7 months (range, 0.5-22) after allo-HSCT. Among all cases of relapse, 20 were hematological relapse, 6 were cytogenetic relapse, while 4 were extramedullary relapse. Meanwhile, in 126 patients receiving G-CSF-mobilized PBSCs, relapse occurred in 39 cases at a median period of 5 months (range, 1-34.5), including 29 hematological relapse, 7 cytogenetic relapse as well as 3 extramedullary relapse.

As for the cumulative incidences of 3-year TRM, there was no obvious difference between G-CSF-mobilized BM and PBSCs cohort and G-CSF-primed PBSCs group (11.5% *vs.* 14.6%, HR, 0.815; 95% CI: 0.402-1.652, *P*=0.570, **Figure 1B**). The proportion of patients with bacterial infections was not remarkably different in the two cohorts (34.4% *vs.* 39.7%, *P*=0.386). The incidence of CMV reactivation in G-CSF-mobilized BM and PBSCs cohort was 71.2%, which was not different from that in G-CSF-primed PBSCs group of 67.5% (*P*=0.521). Due to intensive surveillance and pre-emptive antiviral therapy, no onset of CMV associated diseases was observed in both cohorts.

### Survival and causes of death

For the entire study cohort, 3-year probabilities of OS and DFS were 65.4%, 61.3%, separately. The median follow-up period after allo-HSCT among alive cases between G-CSF-mobilized BM and PBSCs cohort and G-CSF-primed PBSCs group were 39 months (22–91) and 41 months (22–92), respectively (*P*=0.127). As presented in **Figure 1C–D**, 3-year probabilities of OS for patients receiving G-CSF-mobilized BM and PBSCs and G-CSF-primed PBSCs were 69.5% *vs.* 61.4%, respectively (HR, 0.769; 95% CI: 0.502-1.176; *P*=0.226). Furthermore, 3-year probabilities of DFS for patients in two cohorts were 65.4% *vs.* 57.4%, respectively (HR, 0.784; 95% CI: 0.524-1.173; *P*=0.237).

A total of 38 patients in the G-CSF-mobilized BM and PBSCs cohort and 48 patients in the G-CSF-mobilized PBSCs group experienced death during the study process. Recurrence of primary disease was the most frequent reason of death in two groups (24 cases *vs.* 31 cases). In G-CSF-mobilized BM and PBSCs cohort, nine cases died of pneumonia, two of graft failure, one of aGVHD, one of cGVHD, and one of unknown reasons. In contrast, pulmonary infection-related deaths (seven cases) and graft failure-related deaths (six cases) were common seen in the G-CSF-mobilized PBSCs group. One case died of aGVHD, two of cGVHD, and one of intracranial infection. Out of 17 patients who died due to infection, eight cases died before engraftment (5 in the G-CSF-primed BM and PBSCs cohort and 3 in the G-CSF-mobilized PBSCs cohort). **Table 2** delineates causes of death for the two cohorts comprehensively.

**Table 2:** Cause of death after haplo-HSCT in patients who received G-CSF-primed BM and PBSCs mixture or G-CSF-mobilized PBSCs graft.

Cause of death	G-CSF-primed BM and PBSCs group	G-CSF-mobilized PBSCs group
Primary disease	24	31
Pulmonary infection Graft failure	9 2	7 6
aGVHD	1	1
cGVHD	1	2
Intracranial infection Unknown	0 1	1 0

HSCT, hematopoietic stem cell transplantation; G-CSF, granulocyte colony-stimulating factor; BM, bone marrow; PBSCs, peripheral blood stem cells; aGVHD, acute graft-versus-host disease; cGVHD, chronic graft-versus-host disease.

### Multivariate analysis

As shown in **Table 3**, we found no remarkable differences in the relationships between relapse, TRM, survival and the variables including age, gender, donor source, HLA typing, HCT-CI, donor-recipient CMV status, donor-recipient relationship, doses of infused nucleated and CD34+ cells by multivariate analysis. Patients in NR status at transplantation had a remarkably increased relapse, inferior three-year OS and DFS (*P*=0.002, *P*=0.013 and *P*=0.017). Patients having high-risk factors experienced significantly higher relapse, worse three-year OS and DFS (*P*=0.037, *P*=0.016 and *P*=0.014). Patients receiving intensified regimen had reduced relapse, improved OS and DFS (*P*=0.032, *P*=0.028 and *P*=0.027). Occurrence of III-IV aGVHD was prominently correlated with higher TRM, inferior OS and DFS (*P*=0.045, *P*=0.026, *P*=0.042). Patients developing cytomegaloviremia had higher TRM (*P*=0.036), while those with limited cGVHD had better OS and DFS (*P*=0.038, *P*=0.043).

**Table 3:** Multivariate analysis of factors associated with outcomes of patients with hematological malignancies undergoing ATG-based haploidentical stem cell transplantation.

Covariates	HR	95% CI	*P*-value
Relapse			
NR *vs.* CR	2.489	1.298 -5.231	0.002
High risk *vs.* intermediate risk	1.796	1.199-3.926	0.037
intensified regimen *vs.* traditional	0.418	0.181-0.939	0.032
TRM			
Cytomegaloviremia	2.489	1.276-3.896	0.036
III-IV aGVHD	2.287	1.233-3.984	0.045
Overall survival			
NR *vs.* CR	2.731	1.417-4.061	0.013
High risk *vs.* intermediate risk	2.081	1.092-4.231	0.016
intensified regimen *vs.* traditional	0.521	0.259-0.949	0.028
III-IV aGVHD	2.499	1.221-3.962	0.026
Limited cGVHD	0.623	0.317-0.918	0.038
Disease-free survival			
NR *vs.* CR	2.511	1.541-4.159	0.017
High risk *vs.* intermediate risk	2.399	1.125-4.379	0.014
intensified regimen *vs.* traditional	0.532	0.278-0.887	0.027
III-IV aGVHD	2.501	1.242-3.928	0.042
Limited cGVHD	0.541	0.281-0.917	0.043

allo-HSCT, allogeneic hematopoietic stem cell transplantation; NR no remission, CR complete remission, aGVHD acute graft-versus-host disease, cGVHD chronic graft-versus-host disease, HR hazard ratio, CI confidence interval.

### The influence of nucleated cells on transplant outcomes for the entire cohort

**Figure 2:**
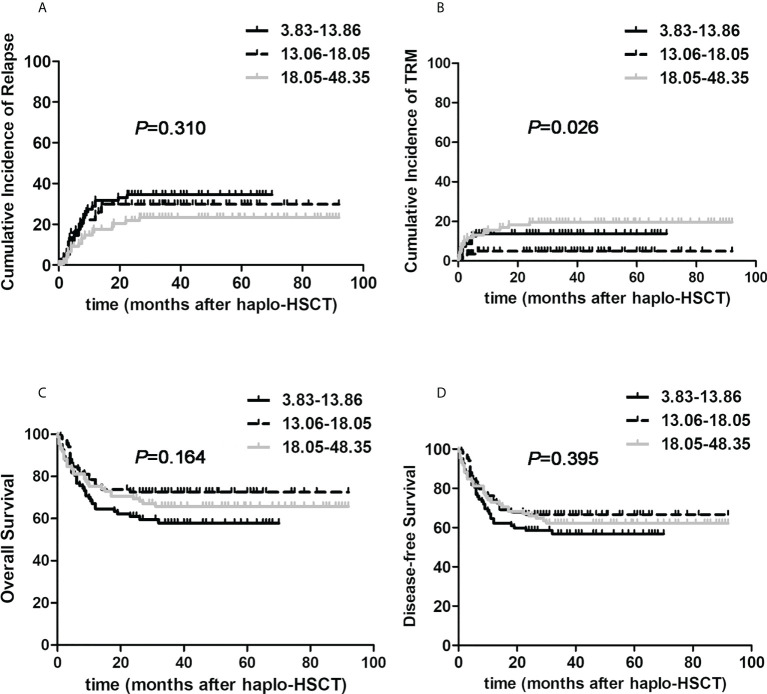
The effects of total nucleated cells on post-transplant outcomes of patients with hematological malignancies undergoing ATG-based haplo-HSCT. The number of infused total nucleated cells was divided into three subgroups according to the number of infused total nucleated cells: 3.83-13.06×10^8^/kg (the black solid line, n=84), 13.06-18.05×10^8^/kg (the black dotted line, n=83), 18.05-48.35×10^8^/kg (the gray solid line, n=84). **(A)** relapse; **(B)** TRM; **(C)** Overall survival; **(D)** Disease-free survival.

We then attempted to explore the influence of graft contents on transplant outcomes in the whole population with hematologic malignancies undergoing haplo-HSCT. Firstly, we divided all cases into three subgroups averagely according to the number of infused TNCs: 3.83-13.06×10^8^/kg, 13.06-18.05×10^8^/kg, 18.05-48.35×10^8^/kg. The three cohorts were comparable in terms of clinical characteristics which were shown in **Supplementary Table 1**. As delineated in **Figure 2**, the 3-year cumulative incidences of relapse were 34.6%, 29.8% and 23.4%, separately (*P*=0.310). Three-year TRM of 13.06-18.05×10^8^/kg group was only 4.9%, which was remarkably reduced when compared to lower and higher groups with corresponding value 18.3%, 19.6% (*P*=0.026). The 3-year OS probabilities were 57.8%, 72.6% and 65.7%, respectively (*P*=0.164). And 3-year DFS probabilities were 56.8%, 66.5% and 61.0%, separately (*P*=0.395). These results shed some light on the slightly improved real-life outcomes of haplo-HSCT through controlling the infused TNCs of harvests. Then, we divided all patients averagely into three groups on the basis of the amount of infused CD34+ cells: 1.65-5.21×10^8^/kg, 5.21-7.60×10^8^/kg, 7.60-25.10×10^8^/kg. Nevertheless, no remarkable correlations between the amount of total CD34+ cells and transplant outcome indicators were found.

### Beneficial role of higher-dose nucleated cells from G-CSF-mobilized BM harvests in haplo-HSCT using G-CSF-mobilized BM and PBSCs mixture

**Figure 3:**
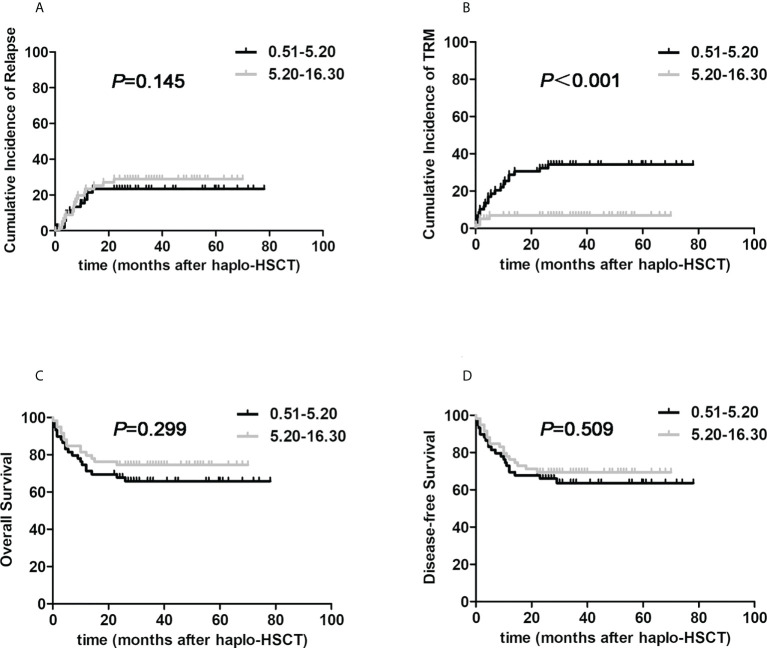
The impacts of nucleated cells from G-CSF-primed BM harvests on post-transplant outcomes in halpo-HSCT using G-CSF-primed BM and PBSCs mixture. The G-CSF-mobilized BM and PBSCs cohort was classified into two groups according to infused nucleated cells harvested from G-CSF-primed BM: 0.51-5.20×10^8^/kg (the black line, n=62), 5.20-16.30×10^8^/kg (the gray line, n=63). **(A)** relapse; **(B)** TRM; **(C)** Overall survival; **(D)** Disease-free survival.

We finally tried to answer which pivotal factor resulted in the substantially better survival benefit in the G-CSF-mobilized BM and PBSCs cohort under the circumstance of haplo-HSCT. Due to the limited number, the G-CSF-mobilized BM and PBSCs cohort was classified into dichotomized groups in terms of median infused nucleated cells or CD34+ cells harvested from G-CSF-primed BM or G-CSF-mobilized PBSCs, respectively. We firstly testified the relationship between transplant outcomes and the amount of nucleated cells in G-CSF-mobilized BM harvest: 0.51-5.20×10^8^/kg, 5.20-16.30×10^8^/kg. The two groups were comparable in terms of clinical characteristics which were shown in **Supplementary Table 2**. As presented in **Figure 3**, 3-year cumulative incidences of recurrence were 28.9% versus 23.4%, respectively (HR, 0.792, 95% CI: 0.376-1.667, *P*=0.145). Encouragingly, 3-year TRM of higher G-CSF-primed BM nucleated cells group was significantly reduced when compared to contemporaneous lower group (6.9% versus 34.2%, HR, 4.058, 95% CI: 1.817-9.006, *P*<0.001). The proportion of patients suffering from infectious events was significantly reduced in the higher G-CSF-primed BM nucleated cells cohort (4.8% *vs.* 29.0%, *P*=0.001). The 3-year OS and DFS probabilities of two cohorts were 74.6% versus 65.8%, 69.5% versus 63.6%, respectively (HR, 1.424, 95% CI: 0.731-2.775, *P*=0.299; HR, 1.237, 95% CI: 0.658-2.326, *P*=0.509). However, it was interesting to note that there were no actually significant correlations between transplantation outcomes and factors including the amounts of G-CSF-primed BM CD34+ cells, nucleated and CD34+ cells in G-CSF-primed PBSCs harvests.

## Discussion

To our best knowledge, the current research represents the first formal comparison of G-CSF-mobilized BM and PBSCs harvests with G-CSF-primed PBSCs only in the context of haplo-HSCT. Our results encouragingly demonstrated that patients with hematologic malignancies receiving G-CSF-mobilized BM integrated with PBSCs harvests in HLA3/6-matched setting had resemble outcomes regardless of relapse, TRM and survival, when compared with patients only receiving G-CSF-primed PBSCs in HLA4/6-matched HSCT. Surprisingly, we proved that the total amount of infused TNCs between 13.06×10^8^/kg and 18.05×10^8^/kg might be the optimal dosage in the specific context of haplo-HSCT. Last but most important, our current study for the first time proved that the infused nucleated cells from G-CSF-primed BM above than 5.20×10^8^/kg could achieve significantly improved NRM in haplo-HSCT being grafted with G-CSF-mobilized BM and PBSCs harvests.

It is now generally acknowledged that G-CSF mobilization is a crucial link during allo-HSCT, because it accelerates cell cycling of pluripotent hematopoietic stem progenitor cells and enhances their amounts in the graft harvests (17). In addition, it transforms the percentage of related cellular subsets as well as their activation status in the BM and PB, inducing functional regulations in donor hematopoiesis and immune system (18, 19). Numerous researches have been conducted in the several past decades to delineate the advantages and disadvantages of each graft source mobilized by G-CSF in populations with hematological malignancies undergoing allo-HSCT (20–22). Couban S and colleagues conducted a phase III, open-label, multicenter randomized study to compare G-CSF-mobilized PBSCs with G-CSF-primed BM in 230 adults with malignant hematological diseases undergoing allo-HSCT using myeloablative conditioning (23). Their studies demonstrates that the usage of G-CSF-primed BM allografts result in a significantly reduced rate of overall cGVHD without losing the graft-versus-leukemia (GVL) effect and approximately considerable OS when compared with G-CSF-mobilized PBSCs. However, this trial mainly focused on HLA-matched sibling transplantation. Xu et al. compared the outcomes of 14 patients with high-risk acute leukemia who received G-CSF-mobilized PBSCs with 109 patients who used combined G-CSF-mobilized BM and PBSCs harvests. Their study indicated that the G-CSF-mobilized PBSCs harvest might be inferior to the G-CSF-mobilized BM and PBSCs harvests because of lower engraftment tendency, higher 2-year TRM, similar rates of 2-year relapse (24). Nevertheless, the two studied cohorts were not contemporaneously matched and the enrolled sample size was relatively small. Encouragingly, our current study was the largest trial which demonstrated that patients with hematologic malignancies receiving G-CSF-primed BM with PBSCs harvests in HLA3/6-matched setting had similar outcomes regardless of relapse, TRM and survival, when compared with patients only receiving G-CSF-mobilized PBSCs in HLA4/6-matched HSCT.

Sufficient amount of donor hematopoietic stem cells is considered pivotal to successful transplantation, especially for patients undergoing haplo-HSCT (25–27). So far, there was no established consensus about the concentration of cellular composition in the transplanted grafts. Han et al. retrospectively analyzed data of 73 children with high-risk leukemia undergoing haplo-HSCT and found that patients receiving higher number of TNCs with >10.13×10^8^/kg showed faster hematopoietic reconstitution and lower TRM (28). A research from European Society for blood and marrow transplantation (EBMT) reported that number of TNCs did not affect the survival endpoint after haplo-HSCT for 414 AML patients, while recipients of >4.96×10^6^/kg CD34+ cells experienced reduced TRM and prolonged survival (29). Nevertheless, our analysis showed that the infused TNCs of 13.06-18.05×10^8^/kg would be the best amount which could minimize TRM while ensuring optimal engraftment and exerting GVL effect, and also suggested that the rationale of “more is better” which has been popularized at many centers might be not necessarily true.

ATG-based haplo-HSCT is developing rapidly around the world, G-CSF-primed donor BM and PBSCs transfusion is also an important part of it, and how to balance the proportion of G-CSF-primed BM and PBSCs is a very critical issue. To our knowledge, our study was the first to explore which source of nucleated cells played a predominant role for prognosis of haplo-HSCT receiving G-CSF-mobilized BM combined with PBSCs harvests. And the results indicated that the transplanted nucleated cells from G-CSF-primed BM above than 5.20×10^8^/kg could remarkably reduce TRM. It could be speculated that BM harvests mobilized by G-CSF comprise abundant mesenchymal stem cells/mesenchymal (stromal) progenitor cells, which might harbor immunoregulatory functions and allow the breakthrough of MHC barriers between donors and recipients (30). De Felice et al. compared the influence of G-CSF mobilization on hematopoietic, mesenchymal, and immune cells derived from BM and PB *in vivo*. They found substantial differences between G-CSF-primed BM and PB regarding to hematopoietic cells and immune cell subsets such as a dramatical increment of the mesenchymal progenitors, regulatory T cells, natural killer cells and the conspicuous rise of mesenchymal cell progenitors was detected in G-CSF-primed BM (31). The cytokine-induced or intrinsic biologic characteristics that were significantly relevant to several post-transplant outcomes should be carefully examined in our cohorts in the future.

In conclusion, we found that donor graft composition was a major determinant for haplo-HSCT. Compared with patients only receiving G-CSF-primed PBSCs as grafts, patients receiving G-CSF-mobilized BM combined with PBSCs harvests had similar outcomes with respect to relapse, TRM and survival. A mixed graft with an optimal number of TNCs between 13.06×10^8^/kg and 18.05×10^8^/kg was associated with better transplant outcomes. Most importantly, we firstly proved that infused nucleated cells from G-CSF-primed BM more than 5.20×10^8^/kg associated with improved TRM in haplo-HSCT being grafted with G-CSF-mobilized BM and PBSCs harvests. Nevertheless, we must acknowledge that our study also had limitations. Key limitations of our study are its retrospective design and single-center sampling which might limit the generalizability and interpretation of our findings. We could not carry out subgroup analyses that might have been definitely instructive in our particular population. Future studies with relatively consistent disease composition and conditioning protocols are needed to validate our results.

## Data availability statement

The raw data supporting the conclusions of this article will be made available by the authors, without undue reservation.

## Ethics statement

The studies involving human participants were reviewed and approved by the Institutional Review Board on Medical Ethics at Tongji Medical College of Huazhong University of Science and Technology. Written informed consent to participate in this study was provided by the participants’ legal guardian/next of kin.

## Author contributions

LX and WS conceived the protocol; RZ analyzed, interpreted the data, and wrote the manuscript; XL, LT, and HY carried out the statistical analysis; HW, YY, and ZZ revised the manuscript, and all authors critically reviewed and approved the final version of the manuscript.

## Funding

The present work was supported and funded by the National Natural Science Foundation of China (No. 81974003, 81900181, 81370668), Key Scientific Research Project of Henan University (No. 20A320021), Joint Co-construction Project of Henan Medical Technology Research Plan (No. 2018020028), Key Science and Technology Project of Henan Province (No. SBGJ202103054), Natural Science Foundation of Hubei Province (No.2020CFB772, 2020CFB790) and Collaborative Innovation Center of Hematology of China.

## Conflict of interest

The authors declare that the research was conducted in the absence of any commercial or financial relationships that could be construed as a potential conflict of interest.

## Publisher’s note

All claims expressed in this article are solely those of the authors and do not necessarily represent those of their affiliated organizations, or those of the publisher, the editors and the reviewers. Any product that may be evaluated in this article, or claim that may be made by its manufacturer, is not guaranteed or endorsed by the publisher.
