# Supplementary material for: Influence of graft composition in patients with hematological malignancies undergoing ATG-based haploidentical stem cell transplantation
Source: Front Immunol. 2022 Sep 15;13:993419. doi: 10.3389/fimmu.2022.993419 (PMC9520486; doi:10.3389/fimmu.2022.993419)
Supplement: Supplementary file 1 [file DataSheet_1.docx]

**Supplementary Table 1** Patient and transplant characteristics in three cohorts according to the number of infused total nucleated cells.

| Characteristics | 3.83-13.06 (×10^8^/kg) | 13.06-18.05 (×10^8^/kg) | 18.05-48.35 (×10^8^/kg) | *P*-value |
| --- | --- | --- | --- | --- |
| No. of patients  Age, median (range), year  Gender  Male  Female  Underlying disease, n (%)  AML  ALL  MDS  Risk classification, n (%)  Standard risk  High risk  HCT-CI, n (%)  0  1-2  ＞2  HLA typing  HLA4/6-matched  HLA3/6-matched Donor/recipient CMV status  Neg/neg  Neg/pos  Pos/neg  Pos/pos  Donor-recipient relationship  Parent donor  Child donor  Sibling donor  Lateral relative donor  Conditioning intensity  Intensified regimen  MAC  Median follow-up for survivors (months, range) | 84  29 (8-56)  51 (60.7%)  33 (39.3%)  45 (53.6%)  28 (33.3%)  11 (13.1%)  22 (29.7%)  52 (70.3%)  30 (35.7%)  29 (34.5%)  25 (29.8%)  40 (47.6%)  44 (52.4%)  38 (45.2%)  16 (19.0%)  16 (19.0%)  14 (16.7%)  36 (42.9%)  14 (16.7%)  34 (40.5%)  0 (0)  63 (75.0%)  21 (25.0%)  38 (22-91) | 83  28 (6-59)  45 (54.2%)  38 (45.8%)  40 (48.2%)  35 (42.2%)  8 (9.6%)  16 (19.3%)  67 (80.7%)  29 (34.9%)  34 (41.0%)  20 (24.1%)  39 (47.0%)  44 (53.0%)  34 (41.0%)  19 (22.9%)  18 (21.7%)  12 (14.5%)  32 (38.6%)  20 (24.1%)  30 (36.1%)  1 (1.2%)  57 (68.7%)  26 (31.3%)  41 (22-92) | 84  29 (9-55)  52 (61.9%)  32 (38.1%)  40 (47.6%)  34 (40.5%)  10 (11.9%)  23 (27.4%)  61 (72.6%)  34 (40.5%)  33 (39.3%)  17 (20.2%)  36 (42.9%)  48 (57.1%)  36 (42.9%)  22 (26.2%)  14 (16.7%)  12 (14.3%)  25 (29.8%)  25 (29.8%)  33 (39.3%)  1 (1.2%)  60 (71.4%)  24 (28.6%)  40 (22-89) | 0.721  0.554  0.780  0.279  0.653  0.798  0.931  0.400  0.661  0.547 |

*AML* acute myeloid leukemia, *ALL* acute lymphoblastic leukemia, *MDS* myelodysplastic syndrome, *HLA* human leukocyte antigen, *MAC* myeloablative conditioning, *G-CSF* granulocyte colony-stimulating factor, *BM* bone marrow, *PBSCs* peripheral blood stem cells, *HCT-CI* hematopoietic stem cell transplantation-comorbidity index, *CMV* cytomegalovirus.
