# Supplementary material for: Influence of graft composition in patients with hematological malignancies undergoing ATG-based haploidentical stem cell transplantation
Source: Front Immunol. 2022 Sep 15;13:993419. doi: 10.3389/fimmu.2022.993419 (PMC9520486; doi:10.3389/fimmu.2022.993419)
Supplement: Supplementary file 2 [file DataSheet_2.docx]

**Supplementary Table 2** Patient and transplant characteristics in two cohorts according to the amount of nucleated cells in BM harvest in the G-CSF-mobilized BM and PBSCs group.

| Characteristics | 0.51-5.20×10^8^/kg | 5.20-16.30×10^8^/kg | *P*-value |
| --- | --- | --- | --- |
| No. of patients  Age, median (range), year  Gender  Male  Female  Underlying disease, n (%)  AML  ALL  MDS  Risk classification, n (%)  Standard risk  High risk  HCT-CI, n (%)  0  1-2  ＞2  HLA typing  HLA4/6-matched  HLA3/6-matched  Donor/recipient CMV status  Neg/neg  Neg/pos  Pos/neg  Pos/pos  Donor-recipient relationship  Parent donor  Child donor  Sibling donor  Lateral relative donor  Conditioning intensity  Intensified regimen  MAC  Median follow-up for survivors (months, range) | 62  29 (8-56)  28 (45.2%)  34 (54.8%)  27 (43.5%)  25 (40.3%)  10 (16.1%)  15 (24.2%)  47 (75.8%)  24 (38.7%)  25 (40.3%)  13 (21.0%)  7 (11.3%)  55 (88.7%)  30 (48.4%)  11 (17.7%)  12 (19.4%)  9 (14.5%)  22 (35.5%)  14 (22.6%)  25 (40.3%)  1 (1.6%)  43 (69.4%)  19 (30.6%)  39 (22-91) | 63  28 (9-55)  34 (54.0%)  29 (46.0%)  34 (54.0%)  22 (34.9%)  7 (11.1%)  12 (19.0%)  51 (81.0%)  26 (41.3%)  23 (36.5%)  14 (22.2%)  11 (17.5%)  52 (82.5%)  28 (44.4%)  15 (23.8%)  11 (17.5%)  9 (14.3%)  28 (44.4%)  12 (19.0%)  22 (34.9%)  1 (1.6%)  50 (79.4%)  13 (20.6%)  38 (22-90) | 0.518  0.325  0.469  0.485  0.908  0.326  0.869  0.791  0.200  0.315 |

*AML* acute myeloid leukemia, *ALL* acute lymphoblastic leukemia, *MDS* myelodysplastic syndrome, *HLA* human leukocyte antigen, *MAC* myeloablative conditioning, *G-CSF* granulocyte colony-stimulating factor, *BM* bone marrow, *PBSCs* peripheral blood stem cells, *HCT-CI* hematopoietic stem cell transplantation-comorbidity index, *CMV* cytomegalovirus.
